# The promoter regions of the Myb-regulated *Adora2B *and *Mcm4 *genes co-localize with origins of DNA replication

**DOI:** 10.1186/1471-2199-8-75

**Published:** 2007-09-06

**Authors:** Holger Gundelach, Daniel Braas, Karl-Heinz Klempnauer

**Affiliations:** 1 Institute for Biochemistry, Westfälische-Wilhelms-Universität Münster, Wilhelm-Klemm-Str. 2, D-48149 Münster, Germany; 2 Howard Hughes Medical Institute, MacDonald Research Laboratories, UCLA, 675 Charles E. Young Drive South, Los Angeles, CA-90095-1662, USA

## Abstract

**Background:**

The retroviral oncogene *v-myb *encodes a transcription factor (v-Myb) which is responsible for the transformation of myelomonocytic cells by avian myeloblastosis virus (AMV). v-Myb is thought to exert its biological effects by deregulating the expression of specific target genes. We have recently demonstrated that the chicken *Gas41 *gene, whose promoter co-localizes with an origin of DNA replication, is a bona fide Myb target gene. Because of this finding we have asked whether other Myb-regulated genes are also associated with DNA replication origins.

**Results:**

We show that the promoter region of the chicken adenosine receptor 2B gene (*Adora2B*), a known Myb-target gene, acts as a DNA replication origin. Furthermore, we have examined known replication origins for the presence of Myb binding sites. We found that the intergenic region between the genes for the minichromosome maintenance 4 protein (*Mcm4*) and the catalytic subunit of DNA-dependent protein kinase (*Prkdc*), whose human counterpart has been identified as a replication origin, contains a number of Myb binding sites. Our data show that this region also acts as an origin of replication in chicken cells. Interestingly, we found that the chicken *Mcm4 *gene is also Myb-regulated.

**Conclusion:**

Our work identifies the chicken *Mcm4 *gene as a novel Myb target gene and presents evidence for the co-localization of two novel origins of DNA replication with Myb-regulated genes. Our work raises the possibility that a fraction of Myb target gene promoters is associated with DNA replication origins.

## Background

The oncogene v-*myb *of avian myeloblastosis virus (AMV) encodes a transcription factor which is responsible for the ability of AMV to transform cells of the myelomonocytic lineage [1,2]. v-*myb *is derived from the chicken c-*myb *gene, which plays a crucial role in the development of the hematopoietic system. c-*myb *is expressed in immature cells of all hematopoietic lineages and is silenced during the terminal differentiation of these cells. It is thought that c-*myb *is part of a genetic switch that allows hematopoietic progenitor cells to choose between alternative fates, such as proliferation, differentiation or apoptosis [2,3].

The proteins encoded by v-*myb *and c-*myb *(v-Myb and c-Myb) bind to the sequence motif PyAAC(G/T)G [4] and control the expression of a number of genes, such as *mim-1 *[5], the lysozyme gene [6], *bcl-2 *[7,8], *tom-1 *[9], *c-kit *[10], *GBX2 *[11], *Pdcd4 *[12], the genes for neutrophil elastase [13] and the A2B adenosine receptor [14] among others. Recent microarray analyses have suggested that Myb actually affects the expression of a much larger set of genes, either by direct binding or by indirect mechanisms [15].

Most of the work aimed at understanding how Myb activates the expression of its target genes has focused on the promoter regions of these genes and has provided a relatively simple picture of how Myb affects gene expression. In many cases, Myb binding sites have been identified in the promoter regions of these genes, suggesting that Myb acts primarily on the promoters of its targets. Recent in-depth analyses of some Myb target genes, however, have suggested a more complex picture in which the activation of certain target genes is mediated by cooperation of additional Myb-responsive enhancer elements with the target gene promoters. For example, the expression of the chicken *mim-1 *gene is controlled by interactions of Myb both with the promoter as well as with a powerful cell-specific enhancer located upstream of the gene [16]. Similarly, in case of the chicken C/EBPβ gene Myb targets the promoter as well as an enhancer located downstream of the gene [17].

Recently, another regulatory DNA element has been mapped close to a Myb-regulated gene. We have shown that chicken *Gas41*, whose promoter region has been known for some time to act as an origin of DNA replication [18,19], is a direct Myb target gene [20]. Other work has shown that the Myb homolog of *Drosophila melanogaster *is involved in the control of the replication origin responsible for chorion gene amplification [21]. These observations raised the question of whether DNA replication origins are also associated with other Myb target genes. Here, we demonstrate that the promoter region of the Myb-regulated chicken adenosine receptor 2B gene acts as an origin of DNA replication. Furthermore, we identify the chicken *Mcm4 *gene as a novel Myb target gene and demonstrate that the *Mcm4 *promoter is associated with a replication origin. Besides identifying two new DNA replication origins in chicken cells and a novel Myb-regulated gene our work suggests that a significant fraction of Myb-regulated genes might be associated with DNA replication origins.

## Results

### The chicken *Mcm4 *gene is a Myb target gene

**Figure 1:**
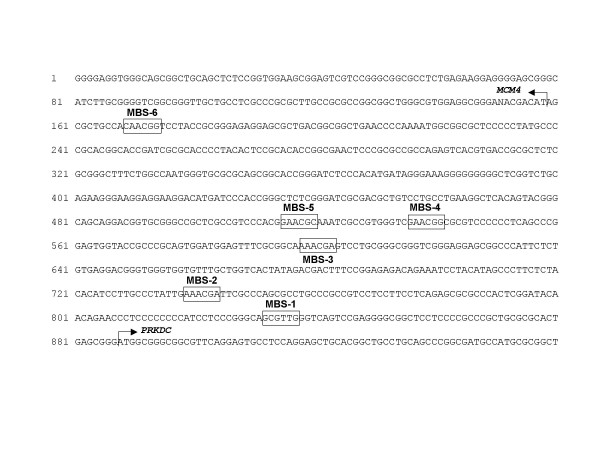
**Nucleotide sequence of the *Mcm4*-Prkdc intergenic region**. The translational start codons of the *Mcm4 *and *Prkdc *genes are marked by arrowheads. Potential Myb binding sites are marked by boxes.

**Figure 2:**
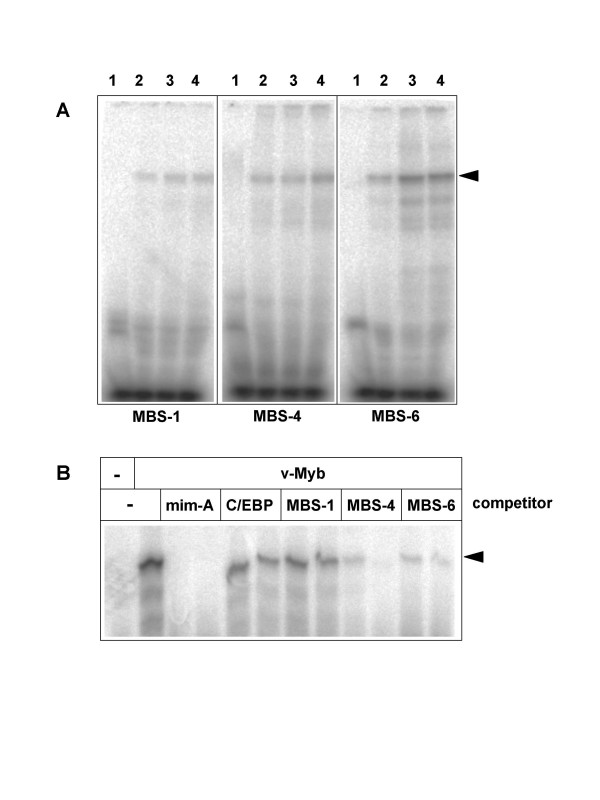
**v-Myb binds to the *Mcm4 *promoter region *in vitro***. **A**. Electrophoretic mobility shift experiments were performed using bacterially expressed full-length v-Myb protein and radiolabeled oligonucleotides corresponding to Myb binding sites MBS-1, MBS-4 and MBS-6. Binding reactions were performed without Myb protein (lanes 1) or with increasing amounts of v-Myb (lanes 2 to 4). Complexes of full-length v-Myb and the oligonucleotides are marked by an arrowhead. The band at the bottom corresponds to unbound oligonucleotides. **B**. Radiolabeled oligonucleotide containing the Myb binding site A of the *mim-1 *promoter were incubated with bacterially expressed full-length v-Myb and excess of unlabeled competitor oligonucleotides, as indicated at the top. Competitors were used at 40-fold or 80-fold molar excess of unlabeled over labeled oligonucleotide, respectively. Only the upper part of the gel containing the complex of v-Myb and the radioactive oligonucleotide is shown.

The observation that the promoter of the Myb-regulated the chicken *Gas41 *gene [20] is associated with an origin of DNA-replication [18,19] prompted us to explore the possibility that other Myb target regions also co-localize with DNA replication origins. Ladenburger et al [22] have previously identified a replication origin located between the human *Mcm4 *and *Prkdc *genes. Both genes are separated from each other by an intergenic region of only 0.8 kb and are transcribed in opposite directions. This arrangement is conserved among humans, mice and chickens [23,24]. We noted that the corresponding chicken sequence contains a number of potential Myb binding motifs (Fig. 1). One of the sites (MBS-6) matched exactly to the consensus Myb binding motif PyAAC(G/T)G [4], while the other sites had one (MBS-4, MBS-1) or two (MBS-2, MBS-3 and MBS-5) mismatches. The human *Mcm4*-*Prkdc *intergenic region also contains 8 potential Myb binding motifs although their exact positions are not conserved between the human and the chicken sequence. To demonstrate that Myb recognizes at least some of the motifs found in the chicken sequence those sites (MBS-1, MBS-4 and MBS-6) that showed the best fit to the consensus binding motif were analyzed by electrophoretic mobility shift assays (EMSA) using recombinant v-Myb protein expressed in bacteria. As illustrated in Fig. 2A, retarded protein-DNA complexes were observed when oligonucleotides containing MBS-1, MBS-4 and MBS-6 were used, demonstrating that v-Myb is able to recognize at least three of the potential binding sites *in vitro*. To confirm the specificity of binding we performed a competition experiment in which binding of v-Myb to a radiolabeled oligonucleotide containing the high affinity Myb binding site A from the *mim-1 *promoter [5] was competed by excess of unlabeled oligonucleotides containing MBS-1, MBS-4 and MBS-6 (Fig. 2B). Binding was competed efficiently by the *mim-1 *A site itself but not by a control oligonucleotide containing a C/EBP binding site. Oligonucleotides containing MBS-4 and MBS-6 also competed, although less efficiently that the *mim-1 *A oligonucleotide. MBS-1 appeared not to compete specifically with the *mim-1 *A site. Taken together, this experiment confirmed that v-Myb binds specifically to binding sites 4 and 6 while binding to MBS-1 was quite weak.

**Figure 3:**
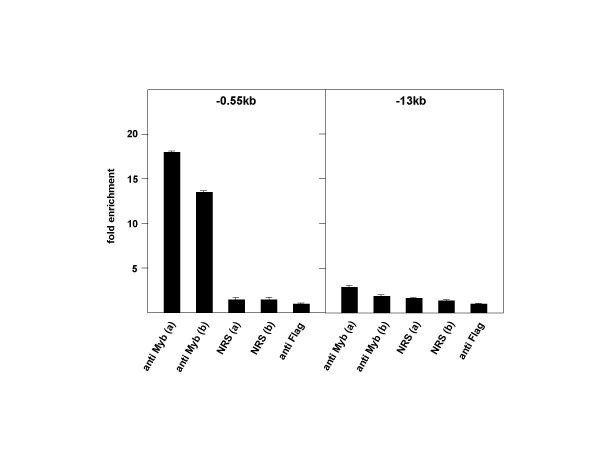
**Chromatin immunoprecipitation of the *Mcm4 *promoter region**. Chromatin fragments prepared from BM2 cells were subjected to immunoprecipitation using two different Myb-specific rabbit antisera raised against the DNA-binding domain of v-Myb (anti Myb a and b). Control immunoprecipitations were performed with two different batches of normal rabbit serum (NRS a and b) or anti-Flag antibodies (anti Flag). DNA isolated from the immunoprecipitates was analyzed by quantitative real-time PCR using primers specific for the *Mcm4 *promoter region (-0.55 kb, left panel) or for a region approximately 13 kb upstream of the *Mcm4 *gene (-13 kb, right panel). The columns indicate the amount of PCR product obtained relative to the anti-Flag control reaction.

To investigate whether v-Myb is bound to the *Mcm4 *promoter region *in vivo *we performed chromatin immunoprecipitation (ChIP) experiments using the v-Myb transformed myeloblast line BM2. The cells were formaldehyde-fixed and DNA-protein complexes obtained after sonication were immunoprecipitated with two different antibodies against Myb. As a control, parallel incubations with non-immune serum or with unrelated antibodies were performed. After reverse-crosslinking and purification of the immunoprecipitated DNA the samples were analyzed by quantitative real-time PCR using the primers that bind to the *Mcm4-Prkdc *intergenic region or control primers. The result of the ChIP experiment is illustrated in Fig. 3. We observed substantial enrichment of the *Mcm4-Prkdc *intergenic region in samples precipitated with the Myb-specific antibodies, whereas control immunoprecipitations with non-immune sera or unrelated antibodies showed no specific enrichment. PCR-reactions with control primers that were specific for sequences located 13 kilobasepairs upstream of *Mcm4 *did not show significant enrichment in any of the immunoprecipitations. Taken together, the ChIP experiment clearly showed that v-Myb was bound to the *Mcm4-Prkdc *intergenic region *in vivo*.

**Figure 4:**
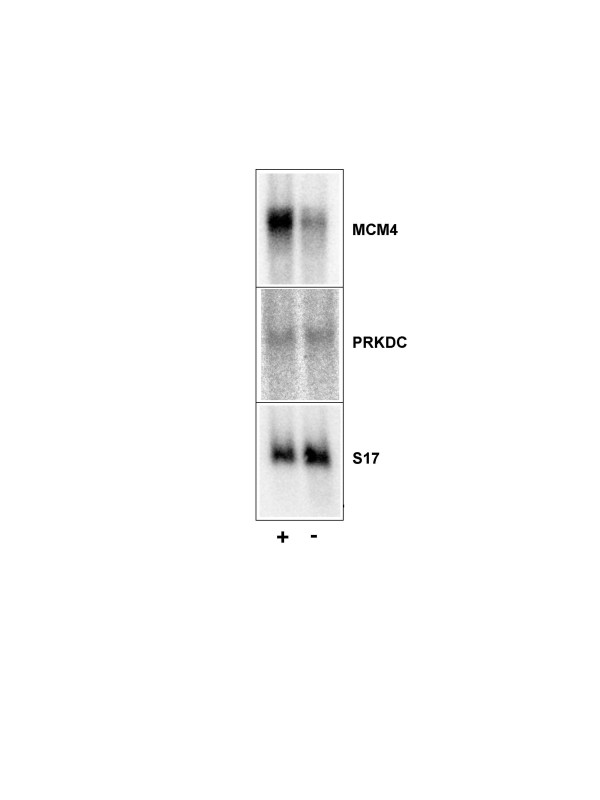
**The chicken *Mcm4 *gene is regulated by v-Myb**. Polyadenylated RNA from 10.4 cells grown for 24 hours in the presence (+ lanes) or absence (- lanes) of 2 μM β-estradiol was analyzed by Northern blotting using probes specific for *Mcm4*, *Prkdc *or *S17 *mRNA.

Next, we investigated whether v-Myb also affected the expression of the *Mcm4 *or *Prkdc *genes. To address this issue we made use of a cell line, designated 10.4, which stably expresses a v-Myb/estrogen receptor fusion protein [25]. Upon β-estradiol treatment of 10.4 cells the v-Myb/ER fusion protein is activated and upregulates the expression of a number of Myb target genes. 10.4 cells were cultivated for 24 hours in the presence or absence of 2 μM β-estradiol. Polyadenylated RNA from these cells was then isolated and analyzed by Northern blotting with probes specific for *Mcm4 *or *Prkdc *mRNA. As a control, a probe hybridizing to the ribosomal protein *S17 *mRNA was used. As shown in Fig. 4, the *Mcm4 *mRNA level was elevated in the estrogen-treated 10.4 cells when compared to untreated cells, suggesting that chicken *Mcm4 *is indeed a Myb target gene. By contrast, *Prkdc *was not regulated by v-Myb. 10.4 cells do not express endogenous estrogen receptor [25], suggesting that *Mcm4 *is not simply an estrogen-regulated gene.

### An origin of DNA replication is located upstream of the chicken *Mcm4 *gene

**Figure 5:**
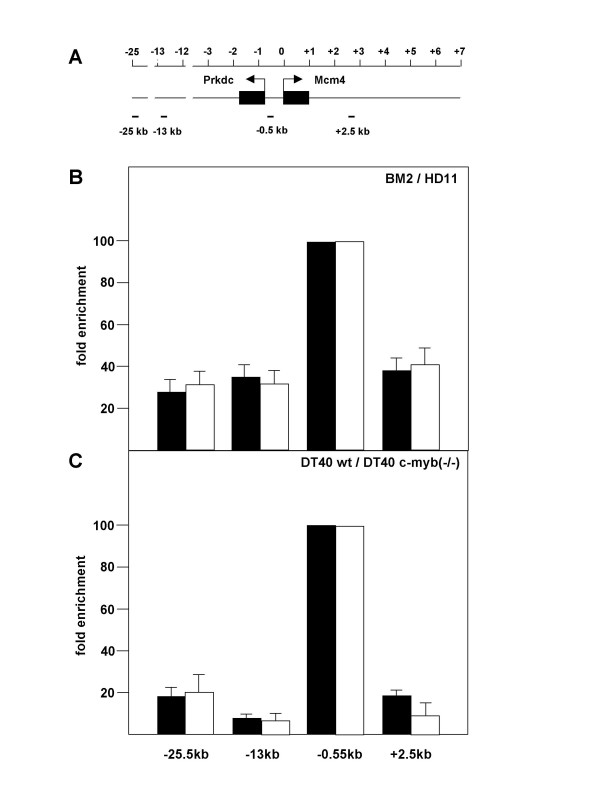
**Replication origin activity of the *Mcm4-Prkdc *intergenic region in chicken cells. A**. Schematic illustration of the *Mcm4-Prkdc *intergenic region. The black boxes and the arrows denote the transcriptional start sites of both genes. The positions of primer pairs used in the PCR analysis are shown below. The scale at the top is in kilobases. **B**. Nascent strand abundance assay. For quantification, real-time PCR was performed using the primer pairs indicated at the bottom. The columns show the average abundance of the corresponding sequences in nascent strand preparations of BM2 (black columns) or HD11 (white columns) cells relative to the sequences amplified with the -0.5 kb primer pair. Error bars mark the standard deviations. **C**. Nascent strand abundance analysis of the *Mcm4 *promoter region using wild-type (black columns) or c-*myb *deficient (white columns) DT40 cells.

To investigate whether the intergenic region between *Mcm4 *and *Prkdc *also acts as a DNA replication origin in chicken cells we performed nascent strand abundance assays as described by Giacca et al. [26]. The nascent strand abundance assay is a powerful and widely used method to identify and map DNA replication origins. The assay is based on the isolation and quantification of single stranded DNA 1–2 kb in length (so-called nascent DNA) which is primarily derived from replication origin regions. The method can be performed with asynchronously growing cells and does not depend on the availability of antisera against origin binding proteins (such as Orc- or MCM-proteins). Briefly, nascent DNA 1–2 kb in length was isolated from different chicken cells by sucrose gradient sedimentation. Sets of primers mapping to the intergenic region as well as to upstream and downstream sequences were used to determine the abundance of the corresponding sequences in nascent DNA using quantitative real-time PCR. BM2 cells are myeloid v-*myb *transformed cells that express high levels of the v-Myb protein. HD11 cells are myeloid cells that do not express v-Myb or c-Myb. DT40 is a line of B-lymphoid cells that express c-Myb. Finally, we used a subclone of the DT40 line in which the c-*myb *gene had been disrupted by homologous recombination [27]. As illustrated in Fig. 5 the *Mcm4-Prkdc *intergenic region was enriched in nascent DNA in each of these cell lines. This demonstrated that this region acts as a DNA replication origin also in chicken cells. We did not observe significant differences in the abundance of the origin region between myeloid cells expressing (BM2) or not expressing (HD11) v-Myb. Although the abundance of the origin region was higher in DT40 cells compared to myeloid cells, there was no significant difference between wild-type and c-Myb deficient DT40 cells. Thus, although Myb binds to the intergenic region and stimulates *Mcm4 *expression, it does not affect the activity of the replication origin.

### Identification of an origin of DNA-replication in the promoter region of the chicken adenosine receptor 2B gene

**Figure 6:**
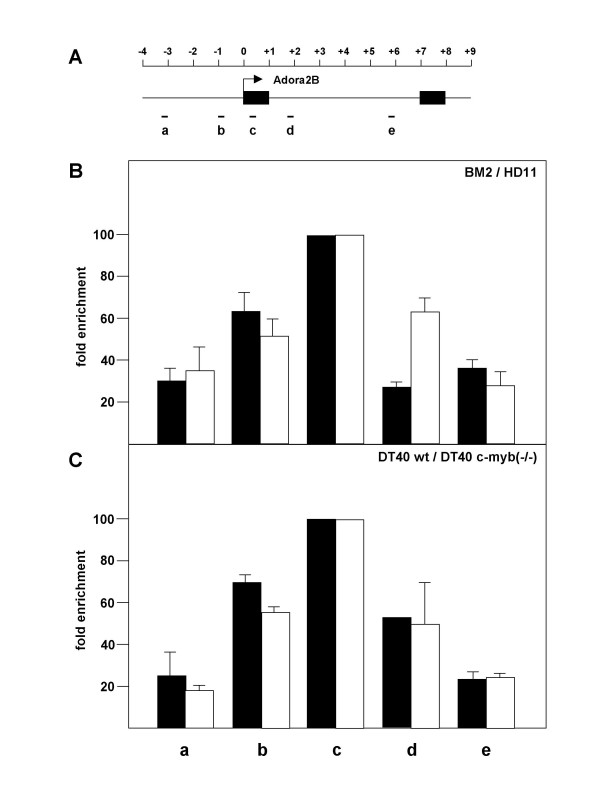
**Replication origin activity of the promoter region of the chicken adenosine receptor 2B gene**. **A**. Schematic illustration of the chicken A2B receptor gene. The black boxes show the two exons of the gene. The transcriptional start site is marked by an arrow. The position of primer pairs used in the PCR analysis is shown below. The scale at the top is in kilobases. **B**. Nascent strand abundance assay of the adenosine receptor 2B promoter region using the PCR primer pairs indicated at the bottom. The columns show the average abundance of the corresponding sequences in nascent strand preparations of BM2 (black columns) or HD11 (white columns) cells relative to the sequences amplified with the -3.9 kb primer pair. Error bars mark the standard deviations. **C**. Nascent strand abundance analysis of the promoter region using wild-type (black columns) or c-*myb *deficient (white columns) DT40 cells.

We also examined the promoters of two known Myb-regulated genes, the *mim-1 *and adenosine 2B receptor genes, for replication origin activity. Both genes are direct Myb targets and their promoters have been analyzed in detail [5,14,28-30]. Nascent DNA from different cell lines was analyzed using sets of primers spanning a region from -5 kb to +5 kb around the promoter in case of the *mim-1 *gene and from -3.2 kb to +4.8 kb around the promoter of the A2B receptor gene. We did not observe enrichment of *mim-1 *promoter sequences in preparations of nascent DNA, indicating the *mim-1 *promoter does not coincide with a DNA replication origin (data not shown). By contrast, the promoter region of the A2B receptor gene was enriched in nascent DNA, suggesting that an origin of DNA-replication is located at the 5' end of the A2B receptor gene (Fig. 6). As in the case of the *Mcm4 *replication origin we did not observe significant differences in origin activity of the promoter region of the A2B receptor gene between the different cell lines.

## Discussion

The identification of the chicken *Gas41 *gene, whose promoter region functions as a DNA replication origin, as a *bona fide *Myb target gene [20] has raised the question whether the co-localization of a Myb-regulated promoter with a replication origin is just a rare coincidence or a more common phenomenon? To address this question we used two different strategies: i.) We examined the promoter regions of known Myb-regulated genes for replication origin activity using the nascent strand abundance assay. ii.) We investigated the activity of v-Myb at the *Mcm4-Prkdc *intergenic region, which harbors several potential Myb binding sites and is associated with a replication origin in human cells [22].

Analysis of the adenosine receptor 2B gene, a known Myb target gene [14,30], showed that the sequences around the 5' end of the gene were enriched in preparations of nascent DNA. This observation strongly suggested that the promoter of the A2B receptor co-localizes with a DNA replication origin. A further case of such co-localization was revealed by the analysis of the *Mcm4-Prkdc *intergenic region. Our data showed that v-Myb binds to this region both in vitro and in vivo and stimulates transcription of the chicken *Mcm4 *gene. Furthermore, we demonstrated that the *Mcm4-Prkdc *intergenic region is a replication origin also in chicken cells. Thus, chicken *Mcm4 *is a novel v-Myb target gene whose promoter region is also associated with a DNA replication origin. As these examples show, Myb-regulated genes and replication origins co-localize in some cases. However, it is also evident from the limited number of genes that have been analyzed so far that not every Myb target gene promoter is associated with a replication origin. For example, the promoter region of the *mim-1 *gene does not possess replication origin activity.

In the light of the co-localization of replication origins with several promoters containing clusters of Myb binding sites we wondered how frequently such clusters occur in the chicken genome. We screened approximately three million basepairs of randomly selected chicken genomic sequences for the presence of clusters of Myb binding motifs (AACG or AACTG) that are similar to those found at the MCM4, Adora2b and Gas41 replication origins. While on the average one such motif is present per 200 base pairs we found that clusters of at least six of such sites within stretches of 250 base pairs occur only once per approximately 25 kb. Although the number of such binding motif clusters is higher than the estimated number of replication origins in the chicken genome (one replication origin per approximately 100 kb, as extrapolated from mammalian cells [31,32]), it appears unlikely that the concurrence of clusters of Myb binding motifs and DNA replication origins reported here is purely accidental. It will therefore be interesting to examine such clusters of Myb binding motifs for their potential role as replication origins.

The functional consequences, if any, of such co-localization are unclear at present. We have not observed that v-Myb or c-Myb influence the activity of the replication origins identified here. v-Myb or c-Myb, therefore, appear not to be involved in the control of origin activity. Recent work on the Myb homolog (Dm-Myb) of *Drosophila melanogaster *has provided evidence that Dm-Myb performs a non-transcriptional role in the control of the replication origin responsible for chorion gene amplification [21]. Furthermore, it has been shown that some of the phenotypic consequences of mutation of Dm-Myb can be compensated by B-Myb, but not by c-Myb or A-Myb [33]. It is therefore conceivable that B-Myb plays a role at the replication origins identified here. Another interesting possibility is that rather than being regulated by Myb the presence of a replication origin might affect the activation of the associated gene by Myb. For example, compacted chromatin structures will be opened during replication thereby providing transcription factors access to the DNA. It is therefore possible that the association with a replication origin imposes an additional level of control onto the adjacent gene, which would be expected to be cell cycle dependent. It is presently not known whether the *Mcm4 *and *Adora2B *genes are expressed in a cell cycle dependent manner. However, in this respect it is interesting to note that the phenotypic changes of myeloid cells transformed by a temperature-sensitive version of v-Myb that accompany activation of v-Myb by down-shifting the cells from the non-permissive to the permissive temperature were not observed when DNA replication was inhibited [34]. This observation is consistent with the idea that the activation of at least some Myb-regulated genes depends on DNA replication. Clearly, further work is necessary until we can discern which of these possibilities, if any, is right.

Independent of a possible role of DNA replication origins in Myb-inducible gene expression our work is also of interest because we have identified two novel replication origins in chicken cells. So far, only very few replication origins are known in chicken cells, such as the origins located in the alpha and beta globin gene clusters [35,36] and between the lysozyme and *Gas41 *genes [18,19]. It is also interesting to note that the origin in the *Mcm4-Prkdc *intergenic region has been conserved between between chickens and humans.

Another interesting aspect of our work is the identification of the chicken *Mcm4 *gene as a Myb-regulated gene. MCM4, together with other MCM proteins, forms the hexameric MCM2-7 complex which functions as helicase during the S-phase of the cell cycle. The MCM2-7 complex is loaded onto the DNA prior to the initiation of DNA replication and constitutes an essential part of the pre-replication complex [for reviews see: [37-39]]. In addition to acting as a helicase in concert with the other MCM proteins forming the MCM2-7 complex there is evidence that MCM4 also performs a specific regulatory role. MCM4 is extensively phosphorylated when cells are exposed to inhibitors of DNA synthesis or to UV irradiation, leading to a block of DNA fork progression [40,41], suggesting that MCM4 plays a role in the DNA replication block checkpoint system. In the normal course of the cell cycle MCM4 is phosphorylated by Cdk2, leading to the inhibition of the helicase function of the MCM complex [42,43]. Blockage of this phosphorylation leads to reinitiation of replication and genomic instability. These observations suggest that MCM4 is a key component of the MCM complex whose manipulation can influence the control of DNA replication and genomic stability. Interestingly, elevated *Mcm4 *mRNA levels have been found in certain tumors, suggesting that increased *Mcm4 *expression can contribute to the malignant properties of the tumor cells [44,45]. Recently, a hypomorph allele of mouse *Mcm4 *was identified and shown to cause chromosomal instability [46]. Thus, *Mcm4 *is an interesting novel Myb target gene that provides a possible link between Myb, DNA-replication and checkpoint control.

## Conclusion

We have identified the chicken *Mcm4 *gene as a novel Myb-regulated gene and shown that the *Mcm4 *promoter region is associated with an origin of DNA replication. Similarly, our work shows that the promoter of the Myb-regulated adenosine receptor 2B gene co-localizes with a replication origin. Our work raises the possibility that a fraction of Myb target gene promoters is associated with DNA replication origins.

## Methods

### Cell culture

The BM2 and HD11 are lines of AMV-transformed chicken myeloblasts and MC29-transformed macrophages that were originally obtained from C. Moscovici and T. Graf, respectively. BM2 cells were grown in RPMI 1640 medium supplemented with 5 % fetal calf serum, 5 % chicken serum and 10 % tryptone phosphate broth (Gibco). HD11 cells were grown in basal Iscoves' medium supplemented with 8% fetal calf serum and 2% chicken serum. 10.4 is a derivative of the HD11 cell line expressing a v-Myb/ER fusion protein [25]. 10.4 cells were grown in the same medium as HD11 cells. To activate the v-Myb/ER fusion protein the growth medium was supplemented with 2 μM β-estradiol for 24 hours.

### Northern blotting

Preparation of polyadenylated RNA and Northern blotting were performed as described [25]. *Mcm4 *and *Prkdc *mRNAs were detected using cDNA clones of chicken *Mcm4 *(riken1_3305r1) and *Prkdc *(riken1_9d19r2), kindly provided by H. Arakawa and J.M. Buerstedde. As an internal control a specific probe for the ribosomal *S17 *gene was used.

### Electrophoretic mobility shift assay

The following pairs of single-stranded oligonucleotides were annealed and used for electrophoretic mobility shift assays:

MCM4-MBS-1_sense: 5'-CCGGGCAGCGTTGGGTCAGTCCGAGGGG-3',

MCM4-MBS-1_anti: 5'-GCCCCTCGGACTGACCCAACGCTGCCCGG-3',

MCM4-MBS-4_sense: 5'-CGCCGTGGGTCGAACGGCGCGTCCCCC-3',

MCM4-MBS-4_anti: 5'-GGGGGGACGCGCCGTTCGACCCACGGCG-3',

MCM4-MBS-6_sense: 5'-TAGCGCTGCCACAACGGTCCTACCG-3',

MCM4-MBS-6_anti: 5'-GCGGTAGGACCGTTGTGGCAGCGCTA-3'.

Mim-1A_sense: 5'-GCTCTAAAAAACCGTTATAATGTACAGATATCTT-3'

Mim-1A_anti: 5'-AAGATATCTGTACATTATAACGGTTTTTTAGAG-3'.

C/EBP_sense: 5'-CTGGCTCGGTTCTTTCACAACCACACATCC-3'

C/EBP_antisense: 5'- GGGATGTGTGGTTGTGAAAGAACCGAGCCAG-3'

After annealing, the oligonucleotides were radiolabeled by filling-in the ends using Klenow polymerase and α^32^P-dCTP. Preparation of bacterial full-length v-Myb protein and binding experiments were performed as described [47].

### Chromatin immunoprecipitation

Approximately 10^8 ^BM2 cells were incubated in 1/10 cross-linking solution containing 0.1 M NaCl; 1 mM EDTA; 0.5 mM EGTA; 50 mM Tris-HCl, pH 8.0 and 11% formaldehyde for 10 min and quenched for 5 min in 125 mM glycine. After washing in ice-cold phosphate-buffered saline, cells were treated for 20 min each with washing solution A (0.25% Triton X100; 10 mM Tris-HCl, pH8.0, 10 mM EDTA; 0.5 mM EGTA) and B (200 mM NaCl; 10 mM Tris-HCl, pH8.0; 1 mM EDTA; 0.5 mM EGTA). Nuclei were sonicated on ice (4 times for 20 sec in 2 min intervals) in egg lysis buffer (ELB) (120 mM NaCl; 50 mM Tris-HCl, pH7.5; 20 mM NaF; 1 mM EDTA; 6 mM EGTA; 15 mM sodiumpyrophosphate; 1 mM phenylmethylsulfonyl fluoride and 0.1% Nonidet P-40). DNA-protein complexes were incubated with two different Myb-specific antibodies raised against the DNA-binding domain of v-Myb [48], normal rabbit serum or anti-Flag antibody (Sigma). After precipitation with protein-A-sepharose the samples were washed several times in ELB buffer. Following elution with 0.5% SDS the DNA was recovered by reverse-crosslinking for 6 h at 37°C and 65°C, respectively, in a buffer containing 0.5% SDS; 10 mM DTT and 100 μg proteinase K. The immunoprecipitated DNA was then purified by phenol-chloroform extraction and ethanol precipitation. PCR-amplification was performed by quantitative real-time PCR using primers specific for the chicken MCM4 promoter region (-0.55 kb) or primers derived from the -13 kb region of the gene.

### Nascent strand abundance assay

Analysis of replication origin activity was performed according to Giacca *et al*. [26]. Total genomic DNA was isolated from approximately 10^8 ^exponentially growing HD11 or BM2 cells using the Nucleobond CB kit (Macherey & Nagel) following the manufacturer's instructions. DNA was resuspended in TNE buffer (10 mM Tris, pH 8.0; 100 mM NaCl; 1 mM EDTA) and heat-denatured for 7 min at 95°C followed by rapid cooling on ice. Denatured DNA was layered on a linear neutral sucrose gradient (5–30% in TNE) and size fractionated by centrifugation in a Beckman SW28 rotor at 26.000 rpm for 20 h at 20°C. Nascent strand DNA fractions with an average DNA size of 1–1.5 kb were pooled, precipitated with ethanol and and treated with T4-polynucleotide kinase and λ-exonuclease as described previously [49,50]. The purified nascent DNA strands were analyzed by quantitative real-time PCR using the following primer pairs:

A2B_-3.2kb_for 5'-TGTCCGCAGAGCAGTTCAG-3';

A2B_-3.2kb_rev 5'-GCAGAACTGGCACAGCTTTCT-3';

A2B_-0.9kb_for 5'-GGTGTTATTGGATAGGCGCTG-3';

A2B_-0.9kb_rev 5'-CACAAAAACATGAGGCAATCG-3';

A2B_+0.4kb_for 5'-CCCATCCCACCTCTCTCGT-3';

A2B_+0.4kb_rev 5'-CACCTTCACCATTGTCGCC-3';

A2B_+1.75kb_for 5'-CCTGGAGTGGGCTTTGACA-3';

A2B_+1.75kb_rev 5'-GCCCTGTTCCCATCCTTGTA-3';

A2B_+4.8kb_for 5'-AAGGTAATGCTGCGACGTCA-3';

A2B_+4.8kb_rev 5'-GTGAAAGCCACACGGAGCTA-3';

MCM4_-13kb_for 5'-CAGCCAGCAAACAGCACTATTTC-3';

MCM4_-13kb_rev 5'-GGCCTTGCTTTCACCTAAGTCA-3';

MCM4_-0.55kb_for 5'-TAGACGACTTTCCGGAGAGACAGA-3';

MCM4_-0.55kb_rev 5'-CGCTGGGCGAATCGTTT-3';

MCM4_+2.5kb_for 5'-CATGGCTGCCAATGAGATCT-3';

MCM4_+2.5kb_rev 5'-GGCCGTACTTGAATTTGATGTTC-3'.

### Real-time PCR quantification

Q-PCR was carried out on a GeneAmp 5700 SDS (Applied Biosystems) with the qPCR Core kit for SYBR Green I (Eurogentec) following the instructions of the manufacturers. Primers were chosen with PrimerExpress 2.0 Software (Applied Biosystems). As DNA standard we used sonicated total genomic chicken DNA (average size 1 to 5 kb) serially diluted to 50.000, 10.000, 2.000, 400 and 80 genomic equivalents per reaction (1 genomic equivalent = 1.25 × 10^-12 ^g). To produce a calibration curve, the C_T _value of each sample was plotted against the logarithm of its concentration. The number of genomic equivalents of each target sequence in the nascent DNA strand samples was then determined by extrapolation from the calibration curve. Several conditions were met by each assayed sample: The slope of the standard curve was around 3 to 3.5 (indicating a PCR efficiency close to 100 %). The sample was diluted such that its genomic equivalents fell within the central part of the calibration curve. The melting curve profile showed a single PCR product. The specificity of the PCR products was additionally confirmed for each primer pair by agarose gel electrophoresis.

## Authors' contributions

HG carried out the in vitro DNA binding and nascent strand abundance assays. DB performed the chromatin immunoprecipitations and northern blot experiments. KHK conceived the study, participated in its design and prepared the manuscript. All authors read and approved the final manuscript.
